# Exploring the oxygenase function of Form II Rubisco for production of glycolate from CO_2_

**DOI:** 10.1186/s13568-021-01224-6

**Published:** 2021-05-08

**Authors:** Fan Yang, Junli Zhang, Zhen Cai, Jie Zhou, Yin Li

**Affiliations:** 1 CAS Key Laboratory of Microbial Physiological and Metabolic Engineering, State Key Laboratory of Microbial Resources, Institute of Microbiology, Chinese Academy of Sciences, Beijing, 100101 China; 2 University of the Chinese Academy of Sciences, Beijing, China; 3 CAS Key Laboratory of Microbial Physiological and Metabolic Engineering, State Key Laboratory of Transducer Technology, Institute of Microbiology, Chinese Academy of Sciences, Beijing, 100101 China

**Keywords:** Rubisco, Oxygenase activity, Glycolate production, Cyanobacteria, CO_2_

## Abstract

The oxygenase activity of Ribulose-1,5-bisphosphate carboxylase/oxygenase (Rubisco) converts ribulose-1,5-bisphosphate (RuBP) into 2-phosphoglycolate, which in turn channels into photorespiration, resulting in carbon and energy loss in higher plants. We observed that glycolate can be accumulated extracellularly when two genes encoding the glycolate dehydrogenase of cyanobacteria *Synechocystis* sp. PCC 6803 were inactivated. This inspired us to explore the oxygenase function of Rubisco for production of glycolate, an important industrial chemical, from CO_2_ by engineered cyanobacteria. Since the oxygenase activity of Rubisco is generally low in CO_2_-rich carboxysome of cyanobacteria, we introduced Form II Rubisco, which cannot be assembled in carboxysome, into the cytoplasm of cyanobacteria. Heterologous expression of a Form II Rubisco from endosymbiont of tubeworm *Riftia pachyptila* (RPE Rubisco) significantly increased glycolate production. We show that the RPE Rubisco is expressed in the cytoplasm. Glycolate production increased upon addition of NaHCO_3_ but decreased upon supplying CO_2_. The titer of glycolate reached 2.8 g/L in 18 days, a 14-fold increase compared with the initial strain with glycolate dehydrogenase inactivated. This is also the highest glycolate titer biotechnologically produced from CO_2_ ever reported. Photosynthetic production of glycolate demonstrated the oxygenase activity of Form II Rubisco can be explored for production of chemicals from CO_2_.

**Supplementary Information:**

The online version contains supplementary material available at 10.1186/s13568-021-01224-6.

## Introduction

Ribulose-1,5-bisphosphate carboxylase/oxygenase (Rubisco) is the key enzyme in photosynthesis (Jensen 2000, Erb et al. 2018). It is responsible for the primary carbon fixation in Calvin-Benson-Bassham (CBB) cycle, catalyzing the addition of CO_2_ to ribulose-1,5-bisphosphate (RuBP), leading to the formation of 3-phosphoglycerate (3PGA) (Moroney et al. 2013). Despite its pivotal role in the biosphere, Rubisco is notorious for its poor carboxylation activity and specificity (Davidi et al. 2020). The poor specificity of Rubisco is due to its oxygenase activity, as CO_2_ and O_2_ are competitive substrates of Rubisco (Moroney et al. 2013). The oxygenation reaction catalyzed by the oxygenase activity of Rubisco results in the production of 2-phosphoglycolate (2PG) (Eisenhut et al. 2008a, b). Although 2PG can be metabolized through photorespiration and recycled back into the central carbon metabolism, this process is energy-consuming and leads to carbon loss (Moroney et al. 2013, Fernie et al. 2020).

The oxygenase activity of Rubisco is often considered undesirable but unavoidable (Moroney et al. 2013). A compelling evidence is that active photorespiration is found in all oxygenic photosynthetic organisms to metabolize 2PG, the toxic oxygenation product of Rubisco (Moroney et al. 2013). Engineering Rubisco for an improved carboxylation efficiency often comes at a price of decreased CO_2_:O_2_ specificity, not to mention the complete removal of its oxygenase activity (Davidi et al. 2020). In fact, there are no CO_2_ or O_2_ binding sites found in Rubisco (Moroney et al.^2013^). Rubisco binds RuBP and converts it to the 2,3-enediol form, allowing the subsequent addition of either CO_2_ or O_2_ (Spreitzer et al. 2002). Due to this catalytic mechanism of Rubisco, it is proposed that the oxygenation reaction of Rubisco cannot be eliminated by mutation (Moroney et al. 2013).

Since the oxygenation function of Rubisco cannot be avoided, and the oxygenation product is involved in the overall carbon metabolism, we propose we can take this advantage to employ the oxygenase activity of Rubisco to produce useful chemicals. In *Synechocystis* sp. PCC 6803 (hereafter *Synechocystis*), 2PG is subsequently converted to glycolate, an important α-hydroxy acid with a wide range of industrial applications in cosmetics, pharmaceuticals and biodegradable polymeric material production (Eisenhut et al. 2006, 2008a, b; Zahoor et al. 2014; Zhan et al. 2020). Especially, the polymer of glycolate (PGA) and the co-polymer of glycolate and lactate (PGLA) are both excellent biodegradable materials with medical applications (Salusjärvi et al. 2017). In engineered heterotrophic cell factories including *Escherichia coli* and *Saccharomyces cerevisiae*, glycolate could be produced from glucose through the glyoxylate shunt or from xylose through xylulose-1-phosohate pathway and xylose oxidation pathway (Koivistoinen et al. 2013; Deng et al. 2015; Alkim et al. 2016; Liu et al. 2018). Recently, a novel glycolate synthetic pathway from glycerol was also successfully constructed in *E. coli* (Zhan et al. 2020). To date, the highest titer of 65.5 g/L with a yield of 0.79 g/g glucose was obtained by balancing the flux distribution between the TCA cycle and glyoxylate shunt in *E. coli* (Deng et al. 2018). However, the production of glycolate from CO_2_ in cyanobacteria has never been reported.

Thus, we intended to produce glycolate from CO_2_ using the oxygenase activity of Rubisco in *Synechocystis*, providing a unique application avenue of the oxygenase activity in photosynthetic biosynthesis.

## Methods and material

### Plasmids and strains construction

All plasmids constructed in this study were summarized in Additional file 1: Table S1. *Escherichia coli* DH5α was used as the host for plasmids construction. All plasmids were generated through Gibson Assembly (NEB, China) of amplified inserts and linearized pUC57 plasmid backbones with primers designed using NEBuilder Assembly Tool (http://nebuilder.neb.com/). All *Synechocystis* mutant strains constructed in this study were summarized in Additional file 1: Table S1. Cyanobacterial strains were generated by transforming cells with certain plasmids which included homologous regions as well as the inserts. Rubiscos were individually overexpressed under the control of the promoter P_cpc560_. The DNA cassette together with a chloromycetin resistance marker was integrated into the *pta* site (slr2132) of *Synechocystis* genome. Transformation of *Synechocystis* was performed as previously described (Lindberg et al. 2010). The colonies were selected on BG-11 plates supplemented with single or combined antibiotics (10 µg/mL chloromycetin, 30 µg/mL erythromycin, 10 µg/mL spectinomycin). Complete segregation and correct gene insertions were checked by PCR and sequencing with primers listed in Additional file 1: Table S2.

### Culture conditions

All strains were grown in 50 mL erlenmeyer flask containing 20 mL of BG11 medium at 30 ^o^C under a constant illumination intensity of 100 µmol photons m^−2^ s^−1^, with atmospheric CO_2_ level or supplemented with prescribed concentration of NaHCO_3_. The initial OD_730_ was normalized to 0.5. Antibiotics were added to the culture for routine maintenance of mutants when necessary. Growth was monitored by measurement of the optical density at 730 nm (OD_730_) every three days.

### Quantification of extracellular glycolate concentration

Extracellular glycolate concentration was determined using the culture supernatant every three days. 10 µL of culture supernatant was analyzed by HPLC equipped with Bio-Rad Aminex® HPX-87H Ion Exclusion Column (300 mm × 7.8 mm) using 8 mM H_2_SO_4_ as mobile phase, pumped at a flow rate of 0.6 mL/min. The column temperature was maintained at 50 °C, and peaks were detected using Agilent Technologies 1260 RID (refractive index detector).

### Quantification of intracellular 2PG and glycolate concentration

The intracellular concentrations of 2PG and glycolate were determined after three days of cultivation. To rapidly quench the cell metabolism, 5 mL of cultures were cooled to 0 ^o^C within 15 s in a −50 ^o^C methanol bath. After centrifugation at 4 ^o^C for 5 min at 8000 × *g*, the cell pellets were washed once with precooled water and resuspended in 2 mL of precooled 80% (vol/vol) methanol solution. After incubation at 20 ^o^C for 30 min, the samples were then centrifuged at 4 ^o^C for 10 min at 20,000 ×*g*. The supernatants were dried by lyophilization and redissolved in 200 µL of water.

The concentrations of 2PG and glycolate were determined with AB Sciex Qtrap 6500 LC-MS/MS System. Injection volume was 5 µL. Metabolites were separated with a HyperREZ XP Organic acid column (100 × 7.7 mm, Thermo Fisher Scientific) with H_2_O as the solvent. The column was maintained at 40 ^o^C with a solvent flow rate of 0.4 mL/min. The electrospray ionization MS was operated in the negative ion mode. The mass spectra was acquired in multiple-reaction monitoring model for the optimized ion pairs of 2PG and glycolate.

### SDS PAGE and Native PAGE

To prepare the protein samples for SDS PAGE and Native PAGE, *Synechocystis* cells were harvested by centrifugation and resuspended with 1 mL buffer (50 mM Tris-HCl, pH 8.0, 10 mM MgCl_2_, 1 mM EDTA) for ultrasonication. After centrifugation, the supernatants were mixed with SDS loading buffer or Native loading buffer at 1:1. The protein samples were detected with SDS PAGE or native PAGE after the total protein amount was normalized to 7 µg.

### Fluorescence microscopy

5 µL log-phase cells were spotted onto 1% (w/v in BG11) agarose pads and air-dried before application of a 0.17 mm coverglass. Fluorescence microscopy was performed on a Nikon N-SIM S Super Resolution Microscope with a 63x/1.4 NA oil-immersion objective using laser lines at 488 nm and 561 nm.

## Results

### Inactivation of two genes encoding glycolate dehydrogenase in *Synechocystis* resulted in glycolate production

**Fig. 1:**
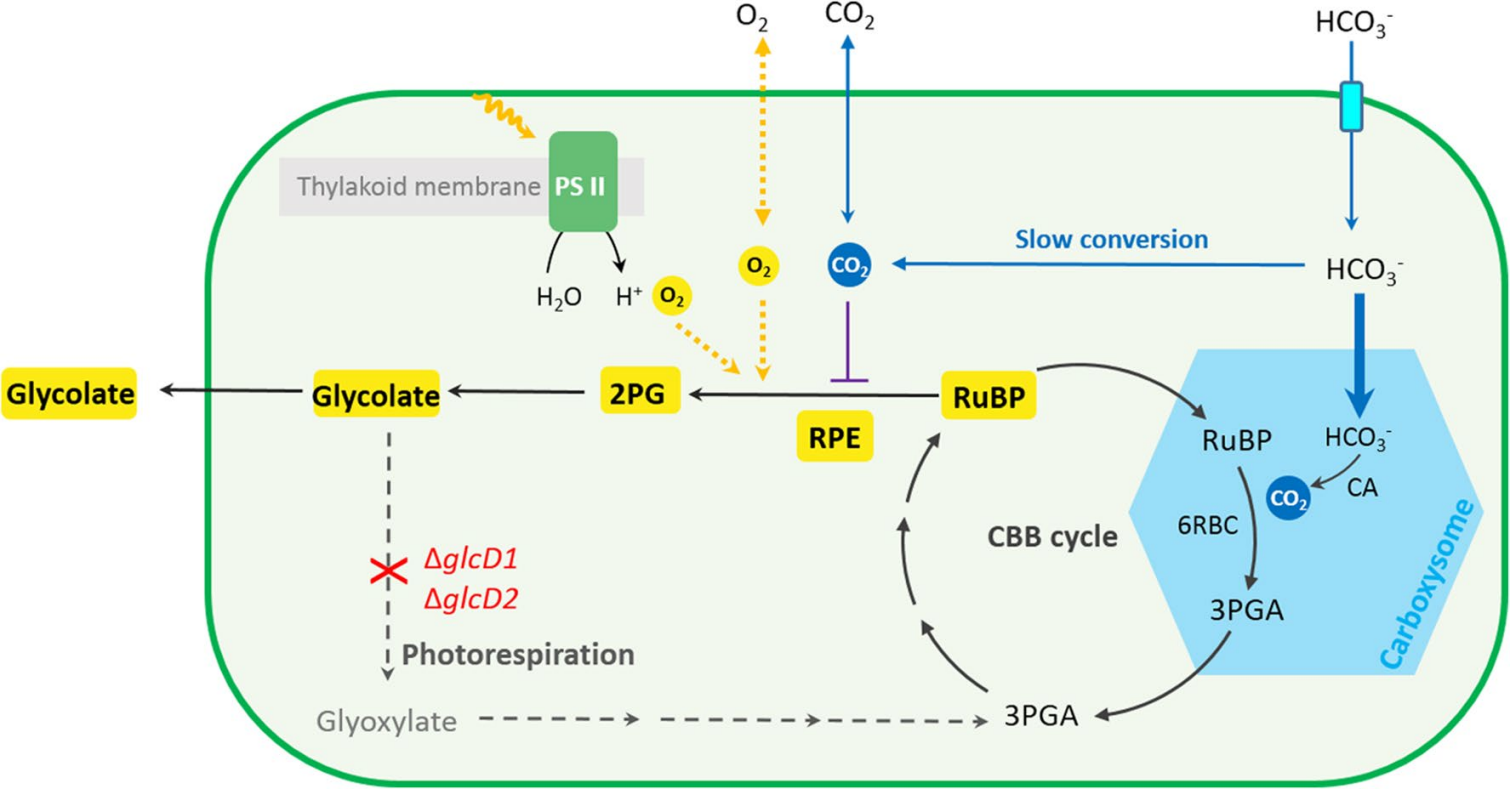
Diagram of glycolate production via oxygenation function of Form II Rubisco in *Synechocystis* sp. PCC 6803. HCO_3_^−^ is actively pumped (light blue) into the cytosol. While some of HCO_3_^−^ spontaneously converts to CO_2_ in cytosol, most of HCO_3_^−^ enters the carboxysome (blue hexagon) and is converted to CO_2_ by the sequestered carbonic anhydrase (CA). RuBP enters the carboxysome and the sequestered Rubisco of *Synechocystis* (6RBC Rubisco) combines RuBP with CO_2_ to generated two molecules of 3-phophoglycerate (3PGA). 3PGA escapes to the cytosol and RuBP is regenerated through the Calvin-Benson-Bassham (CBB) cycle. Photorespiration (gray dashed arrows) can be blocked by inactivating (red cross) two glycolate dehydrogenases (GlcDs) encoded by *glcD1* and *glcD2*, respectively. The resulting strain WT-ΔglcD accumulates and excretes glycolate to the culture (yellow). Form II Rubisco from the endosymbiont of *Riftia pachyptila* (RPE Rubisco) was overexpressed in strain WT-ΔglcD and located in the cytosol. As cyanobacteria performs oxygenic photosynthesis, RPE Rubisco catalyzes the oxygenation of RuBP to 2-photoglycolate (2PG), facilitating glycolate production. As CO_2_ can freely diffuse to the cytosol (blue solid arrow), the elevated CO_2_ level inhibits the oxygenase function of RPE (purple solid line) and decreases glycolate production when the external inorganic carbon is supplied with CO_2_

In *Synechocystis*, glycolate is converted to glyoxylate by two glycolate dehydrogenases (GlcD1 and GlcD2), and subsequently metabolized by three branched routes (Eisenhut et al. 2006, Eisenhut M, Ruth W Eisenhut et al. 2008a, b). To completely block the glycolate metabolism, both GlcD1 and GlcD2 encoded by *glcD1* and *glcD2*, respectively, were inactivated (Fig. 1). The resulting mutant was designated as WT-ΔglcD (Table 1). Complete segregation and correct gene insertions at both *glcD1* and *glcD2* sites were verified by PCR and sequencing (Additional file 1: Fig. S1).

**Table 1:** The *Synechocystis* strains used in this study

Strain	Genetic background	Source of Rubisco
Wild type	*Synechocystis* sp. PCC 6803	–
WT-ΔglcD	WT Δ*glcD1*::*em*^*r*^; Δ*glcD2*::*spec*^*r*^	–
RPE-ΔglcD	Δ*glcD* Δ*pta*::P_cpc560_-*rpe*-T_rbcs_-*cm*^*r*^	*Riftia pachyptila* endosymbiont
4Pm-ΔglcD	Δ*glcD* Δ*pta*::P_cpc560_-*4 pm*-T_rbcs_-*cm*^*r*^	*Phaeospirillum molischianum*
5St-ΔglcD	Δ*glcD* Δ*pta*::P_cpc560_-*5st*-T_rbcs_-*cm*^*r*^	*Sedimenticola thiotaurini*
6Rbc-ΔglcD	Δ*glcD* Δ*pta*::P_cpc560_-*6rbcL*-*6rbcS*-T_rbcs_-*cm*^*r*^	*Synechocystis* sp. PCC 6803

**Fig. 2:**
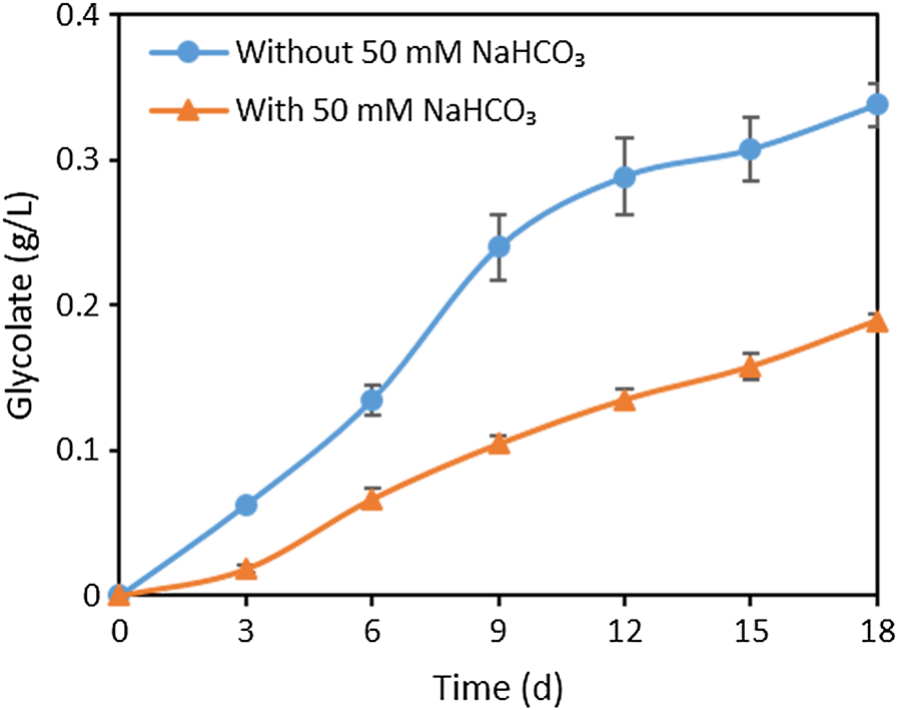
Glycolate production by strain WT-ΔglcD with or without supply of 50 mM NaHCO_3_. The cells were cultivated at 30 ^o^C under 100 µmol photons m^−2^ s^−1^ light intensity. Error bars represent standard deviations from biological triplicates conducted in three independent experiments

As glycolate metabolism was completely blocked, we next investigated glycolate accumulation in strain WT-ΔglcD. Both the intracellular and extracellular glycolate concentrations of WT-ΔglcD were analyzed and compared with that of the WT strain. Samples were taken after three days cultivation supplemented with or without 50 mM NaHCO_3_. The intracellular glycolate concentration of the WT strain was 0.004 µmol L^−1^OD_730_^−1^ and 0.02 µmol L^−1^OD_730_^−1^ respectively, when supplemented with or without 50 mM NaHCO_3_ (Additional file 1: Fig. S2). Moreover, the extracellular glycolate concentration was undetectable in the WT strain under both conditions (data not shown). It is evident that glycolate could be rapidly metabolized in the WT strain. On the contrary, strain WT-ΔglcD accumulated glycolate intracellularly and extracellularly under both conditions (Fig. 2 and Additional file 1: Fig. S2). The intracellular glycolate concentration of strain WT-ΔglcD was 0.51 µmol L^−1^OD_730_^−1^ when supplied with 50 mM NaHCO_3_, and increased to 1.75 µmol L^−1 ^OD_730_^−1^ without the supply of NaHCO_3_ (Additional file 1: Fig. S2). Furthermore, the glycolate concentration in the medium of strain WT-ΔglcD reached 86.47 µmol L^−1^OD_730_^−1^ (mass concentration of 0.02 g/L) and 317.77 µmol L^−1^OD_730_^−1^ (mass concentration of 0.06 g/L) after 3 days cultivation respectively, with or without 50 mM NaHCO_3_ (Fig. 2). Apparently, the majority of glycolate was excreted to the culture by strain WT-ΔglcD, and the intercellular glycolate accumulation could be negligible. We further monitored the glycolate concentration in the medium every three days and found that strain WT-ΔglcD produced 0.19 g/L and 0.34 g/L of glycolate after 18 days cultivation respectively with or without the supply of 50 mM NaHCO_3_ (Fig. 2). In other words, glycolate can be produced from CO_2_ and secreted extracellularly upon inactivation of the two glycolate dehydrogenases in *Synechocystis*. Moreover, strain WT-ΔglcD produces higher concentration of glycolate when no additional NaHCO_3_ was supplemented, suggesting ambient level CO_2_ is sufficient for glycolate production to occur.

### Overexpression of the native carboxysome-located Rubisco didn't contribute to glycolate production

Given the multiple industrial applications of glycolate, we were encouraged to further increase glycolate production. Glycolate synthetic pathway comprises two reactions (Fig. 1). RuBP reacts with O_2_ to generate one molecule of 2PG and one molecule of 3-phosphoglycerate (3PGA) (Eisenhut et al. 2008a, b, Fernie and Bauwe 2020). 2PG is then dephosphorylated to glycolate and 3PGA enters the CBB cycle to regenerate RuBP (Eisenhut et al. 2008a, b, Fernie and Bauwe 2020). In order to identify the bottleneck of glycolate production, the intercellular 2PG concentrations in the WT strain and strain WT-ΔglcD were measured. Samples were taken after three days cultivation with or without the supply of 50 mM NaHCO_3_. With the intact glycolate metabolism, the intracellular 2PG concentration in the WT strain was below 0.03 µmol L^−1^OD_730_^−1^ under both growth conditions (Additional file 1: Fig. S2). The intracellular 2PG level in strain WT-ΔglcD was at the same level as compared to the WT strain. However, as mentioned above, the intracellular glycolate concentration in strain WT-ΔglcD became about 100-fold higher than that of the WT strain irrespective of the supply of 50 mM NaHCO_3_ (Additional file 1: Fig. S2). This indicated that the conversion from 2PG to glycolate in strain WT-ΔglcD was efficient, which is in line with the discovery that up to four 2-phosphoglycolate phosphatases (PGPase) were identified in *Synechocystis* to catalyze this reaction (Rai et al. 2018). Thus, the oxygenation of RuBP catalyzed by Rubisco was the rate-limiting step of glycolate production.

**Fig. 3:**
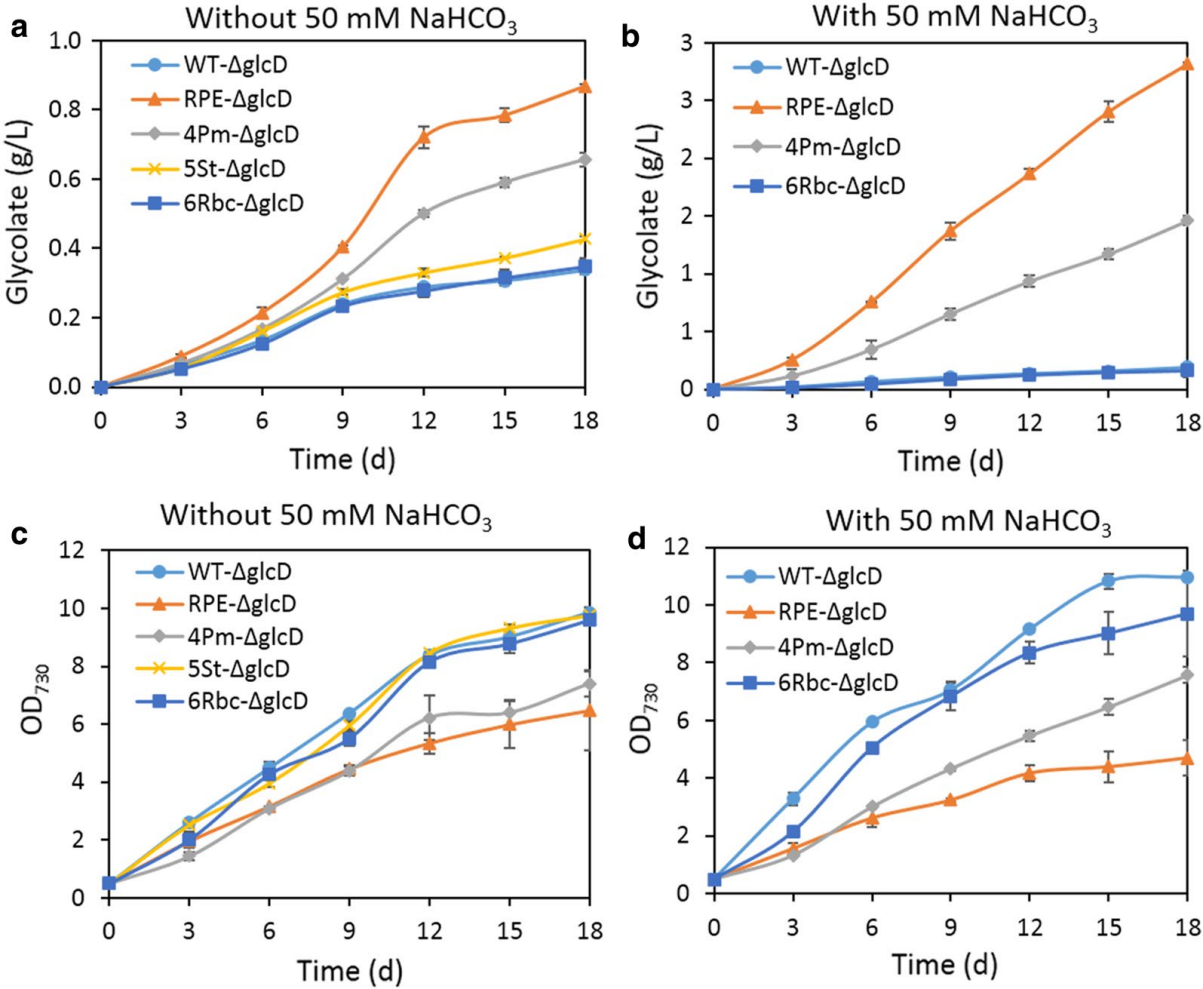
Overexpression of Form II Rubisco increased glycolate production. Glycolate production (**a** and **b**), growth curve (**c** and **d**) of the *Synechocystis* strains expressing different Form II Rubiscos. The cells were cultivated without or with 50 mM NaHCO_3_ at 30 ^o^C under 100 µmol photons m^−2^ s^−1^ light intensity. Error bars represent standard deviations from biological triplicates conducted in three independent experiments

Thus, to increase glycolate production, the native Rubisco of *Synechocystis* was overexpressed in strain WT-ΔglcD. The resulting mutant was designated as strain 6Rbc-ΔglcD (Table 1) and its capacity for glycolate production was determined with the same growth conditions as mentioned above. After 18 days of cultivation, strain 6Rbc-ΔglcD produced 0.16 g/L and 0.35 g/L of glycolate when supplied with or without 50 mM NaHCO_3_, respectively. Neither titer is significantly higher than that of strain WT-ΔglcD under the same condition (Fig. 3a and b). In addition, no significant difference was observed in the growth rates of strains 6Rbc-ΔglcD and WT-ΔglcD under both conditions (Fig. 3c and d). Moreover, the SDS PAGE and native PAGE results suggested that 6RBC was successfully overexpressed and assembled under both conditions (Additional file 1: Fig. S4). These results together suggested that overexpression of 6RBC Rubisco did not contribute to increase glycolate production. The reason behind is likely that the native 6RBC Rubisco is encapsulated in a microcompartment found in all cyanobacteria, termed as the carboxysome. It reduces the oxygenase activity of Rubisco by inhibiting the entrance of O_2_ and increasing CO_2_ concentration around Rubisco (Espie et al. 2011). Thus, to increase glycolate production, the selected Rubisco is expected to be located outside the carboxysome so as its oxygenase activity can play a role.

### Overexpression of Form II rubiscos enhanced glycolate production

It was previously reported that replacing the native Rubisco of cyanobacteria with Form II Rubisco could not support the biogenesis of carboxysome, indicating Form II Rubisco resides outside the carboxysome (Baker et al. 1998; Durao et al. 2015). If the Rubisco is located in the cytosol, it is accessible to molecule oxygen and a reduced CO_2_ level due to the absence of carbonic anhydrase in the cytosol (Price et al. 2008; Price 2011). Thus, we hypothesized that Form II Rubiscos might be promising candidates to increase glycolate production. To this end, three Form II Rubiscos from *Riftia pachyptila* endosymbiont (RPE Rubisco), *Phaeospirillum molischianum* (4Pm Rubisco) and *Sedimenticola thiotaurini* (5St Rubisco) were selected and individually overexpressed by using the strong promoter P_cpc560_ in strain WT-ΔglcD (Table 1), resulting in strains RPE-ΔglcD, 4Pm-ΔglcD and 5St-ΔglcD, respectively (Additional file 1: Fig. S1).

Subsequently, glycolate production of these three strains were determined without additional NaHCO_3_, which seemed to be more favorable for strain WT-ΔglcD to produce glycolate. After 18 days of cultivation, strain 5St-ΔglcD produced 0.43 g/L glycolate, which is not significantly higher than that of strain WT-ΔglcD (Fig. 3a). Moreover, no significant difference on growth was observed between them (Fig. 3c). This incapacity for increasing glycolate production could be attributed to the undetectable expression and assembly of 5St Rubisco (Additional file 1: Fig. S4a). In contrast, glycolate production were dramatically enhanced in strains RPE-ΔglcD and 4Pm-ΔglcD (Fig. 3a). After 18 days of cultivation, strain 4Pm-ΔglcD produced 0.66 g/L of glycolate, about twofold of strain WT-ΔglcD, while strain RPE-ΔglcD produced 0.87 g/L of glycolate, 2.6-fold of strain WT-ΔglcD (Fig. 3a). However, the growth of strains RPE-ΔglcD and 4Pm-ΔglcD were significantly impaired (Fig. 3c). The expression and assembly of RPE Rubisco and 4Pm Rubisco were also detected (Additional file 1: Fig. S4a). RPE Rubisco was copiously overexpressed and well assembled. By contrast, 4Pm Rubisco was successfully overexpressed but not assembled well. This explained their different capacity on enhancement of glycolate production. Taken together, these results showed that overexpression of Form II Rubisco indeed increased glycolate production.

### Supply of NaHCO_3_ increased glycolate production by strains RPE-ΔglcD and 4Pm-ΔglcD

As mentioned above, glycolate production by strain WT-ΔglcD decreased when supplied with 50 mM NaHCO_3_ (Fig. 2). Thus, we further investigated whether glycolate production of strains RPE-ΔglcD and 4Pm-ΔglcD would also be repressed when supplied with 50 mM NaHCO_3_.

Surprisingly, glycolate production by strains RPE-ΔglcD and 4Pm-ΔglcD were not decreased, but instead sharply increased when NaHCO_3_ was available (Fig. 3b). Strain 4Pm-ΔglcD produced 1.46 g/L of glycolate in 18 days when supplemented with 50 mM NaHCO_3_, which is about 7.7-fold of the titer of strain WT-ΔglcD under the same condition (Fig. 3b). This is also more than twofold of the titer produced by strain 4Pm-ΔglcD without additional NaHCO_3_. Additionally, 4Pm Rubisco assembled better upon the addition of 50 mM NaHCO_3_, which could contribute to the increased glycolate production of strain 4Pm-ΔglcD (Additional file 1: Fig. S4b). Among these three strains, strain RPE-ΔglcD was inarguably the best glycolate producer, generating 2.82 g/L after 18 days of cultivation, about 15-fold of the titer of strain WT-ΔglcD under the same growth condition (Fig. 3b). Moreover, the expression and assembly of RPE did not differ upon the addition of NaHCO_3_ (Additional file 1: Fig. S4b), suggesting that the increased glycolate production was not related to the assembly of RPE Rubisco. However, the growth of strains RPE-ΔglcD and 4Pm-ΔglcD were also significantly impaired under this condition (Fig. 3d).

**Fig. 4:**
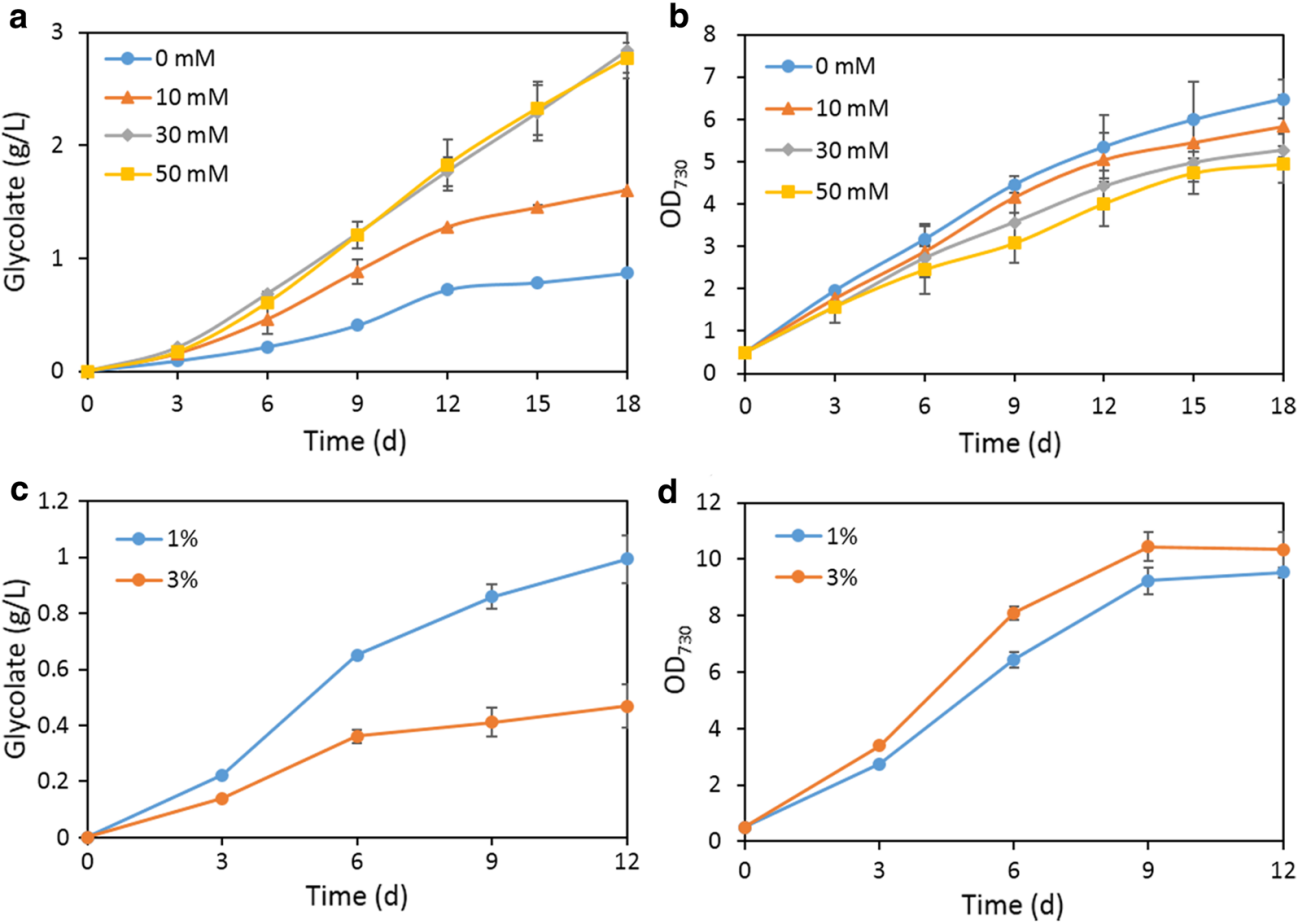
Glycolate production and growth profile of strain RPE-ΔglcD with supply of NaHCO_3_ or CO_2_. Cells were cultivated with different concentrations of NaHCO_3_ (**a** and **b**) or CO_2_ (**c** and **d**) at 30 ^o^C under 100 µmol photons m^−2^ s^−1^ light intensity. Error bars represent standard deviations from biological triplicates conducted in three independent experiments

Thus, we further investigated glycolate production of strain RPE-ΔglcD when supplied with different concentration of NaHCO_3_. Glycolate production of strain RPE-ΔglcD increased along with increasing the concentration of NaHCO_3_, and approached a plateau of 2.84 g/L when supplied with 30 mM NaHCO_3_ (Fig. 4a). Notably, the growth of strain RPE-ΔglcD gradually reduced along with the increased glycolate production (Fig. 4b). The intracellular glycolate concentration in RPE-ΔglcD was also increased, from 5.6 µmol L^−1^OD_730_^−1^ in the absence of NaHCO_3_, to 10.4 µmol L^−1^OD_730_^−1^ when adding 50 mM NaHCO_3_ (Additional file 1: Fig. S2). It was previously reported that intracellular accumulation of glycolate is toxic to the cell (Eisenhut et al. 2008). The retarded growth of strain RPE-ΔglcD upon adding increased concentration of NaHCO_3_ was probably related to the elevated intracellular glycolate concentration.

### Supply of CO_2_ decreased glycolate production by strain RPE-ΔglcD

Cyanobacteria can use both HCO_3_^−^ and CO_2_ as external inorganic carbon source (Price et al. 2008, Price 2011). As supply of HCO_3_^−^ increased glycolate production of strains RPE-ΔglcD and 4Pm-ΔglcD, we then wondered what would be the effect if supplying CO_2_. Since strain RPE-ΔglcD produced much higher titer of glycolate than strain 4Pm-ΔglcD, we chose strain RPE-ΔglcD to study the effect of CO_2_.

To this end, the external organic carbon supplied was changed from NaHCO_3_ to CO_2_. The glycolate production and growth of strain RPE-ΔglcD were evaluated under 1% or 3% CO_2_ (Fig. 4c and d). After 12 days of cultivation, strain RPE-ΔglcD produced 0.87 g/L glycolate under 1% CO_2_, and the glycolate titer decreased to 0.47 g/L under 3% CO_2_ (Fig. 4c). Additionally, the growth of strain RPE-ΔglcD increased positively with increasing the CO_2_ level (Fig. 4d). The increased growth and reduced glycolate production of RPE-ΔglcD together indicated that supply of CO_2_ enhanced the carboxylation reaction of RPE and consequently inhibited the oxygenation reaction.

### RPE rubisco is located in the cytosol

The enhanced glycolate production indicated the active oxygenation reaction catalyzed by RPE Rubisco and 4Pm Rubisco. This suggested that they are probably located in the cytosol rather than in the carboxysome as the O_2_ concentration in cytosol is much higher. To provide direct evidence, we visualized their locations *in vivo* by fluorescent labelling. We first tried to carry out the co-localization analysis by labelling RPE Rubisco with cyan fluorescent protein (CFP) and 6RBC with yellow florescent protein (YFP). RPE Rubisco was labelled with CFP at its C-terminal (termed as RPE-CFP). YFP was fused to the C-terminal of the large subunit of 6RBC (termed as 6RBCL-YFP). RPE-CFP and 6RBCL-YFP were individually expressed in the WT strain to give single fluorescent signal and co-expressed in the WT strain to test whether these two fluorescent signals could be overlayed together. However, the fluorescent signals of RPF-CFP and 6RBCL-YFP were too week to give the location information (data not shown).

**Fig. 5:**
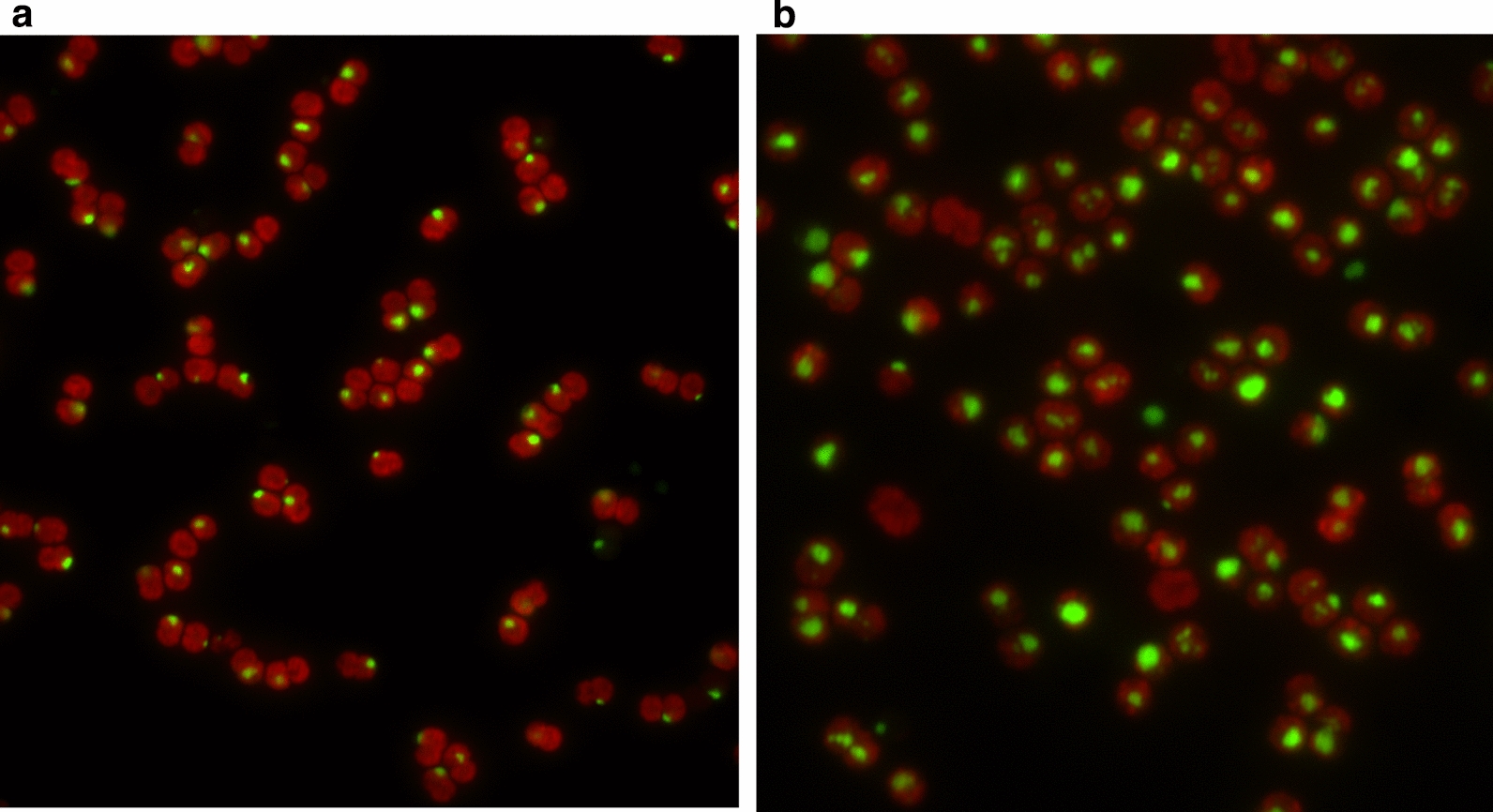
Location of RPE Rubisco in the *Synechocystis* strain. The fluorescent signal of RPE-GFP (**a**) and 6RBCL-GFP (**b**). GFP was fused to the C-terminal of RPE Rubisco or the large subunit of 6RBC Rubisco. RPE-GFP and 6RBCL-GFP were individually expressed in the WT strain. The foci of 6RBC-GFP represents the location of mature carboxysomes. The red fluorescence of endogenous chlorophyll-a was used to indicate the shape of the whole cell

We next fused green fluorescent protein (GFP) to the C-terminal of RPE Rubisco or the large subunit of 6RBC Rubisco, termed as RPE-GFP and 6RBCL-GFP, respectively. RPE-GFP and 6RBCL-GFP were individually expressed in the WT strain to give single fluorescent signal. Meanwhile, the red fluorescence of endogenous chlorophyll-a of *Synechocystis* was used to indicate the shape of the whole cell (Cameron et al. 2013). RPE-GFP gave rise to a large single fluorescent punctum at the cell polar, suggesting that RPE proteins intended to aggregate at the edge of cell (Fig. 5a). By contrast, 6RBCL-GFP intended to exhibit several fluorescent spots at a more central position within the cell, indicating the locations of mature carboxysomes, which was in agreement with the previous report (Fig. 5b) (Cameron et al. 2013). The different positions of fluorescent signals between RPE-GFP and 6RBCL-GFP indicated that RPE is not located in the carboxysome where 6RBCL-GFP resides. The bacterial Form II Rubisco from *Rhodospirillum rubrum* was previously expressed in the Δrbc strain of *Synechocystis* (Durao et al. 2015). The resulting mutant could not support the biogenesis of carboxysome and photoautotrophic growth at ambient CO_2_ concentration (Durao et al. 2015). Thus, it is conceivable that the aggregate of RPE-GFP observed here is most likely in the cytosol.

## Discussion

The oxygenase function of Rubisco and the ensuing photorespiration have long been regarded as one of the obstacles to improve the photosynthesis efficiency (South et al. 2018; Hu et al. 2019; Luan et al. 2020). Cumulative studies have attempted to inhibit even avoid the occurrence of the oxygenation reaction of Rubisco but gained limited progress (Erb and Zarzycki 2018, Davidi et al. 2020). Here, as the oxygenation product of Rubisco is involved in the overall carbon metabolism, we utilized the oxygenation activity of Form II Rubisco for production of glycolate, a versatile chemical with extensive industrial applications, from CO_2_ in *Synechocystis*.

In *Synechocystis*, glycolate can only be generated from 2PG, the direct product of the oxygenation reaction of Rubisco. Glycolate is then converted to glyoxylate and subsequently metabolized by three branched routes including the plant-like photorespiratory cycle, the bacterial glycerate pathway and the complete decarboxylation of glyoxylate to CO_2_ (Eisenhut et al. 2008a, b). In the first instance, glycolate production was primarily achieved by inactivation of two forms of glycolate dehydrogenases which are responsible for converting glycolate to glyoxylate. As glycolate metabolism is completely inactivated, the resulting strain WT-ΔglcD produced glycolate irrespective of the provision of NaHCO_3_. This indicated that Rubisco is performing the oxygenase function despite the active CO_2_-concentrating mechanism (CCM) and the abundance of inorganic carbon, which is also proved in the earlier studies (Eisenhut et al. 2006, Eisenhut et al. 2008a, b). It is still under discussion whether cytosolic Rubisco, which is in the various stages of assembly during carboxysome biogenesis, is responsible for this oxygenase activity, or whether significant amounts of O_2_ indeed enter the carboxysome (Espie and Kimber 2011, Burnap et al. 2015). Since overexpression of 6RBC Rubisco showed no effect on glycolate production, it is conceivable that the availability of O_2_ is limited in carboxysome.

Additionally, inactivation of glycolate metabolism was reported to render a high-CO_2_-requiring (HCR) phenotype which means the mutant was not able to grow at ambient CO_2_ level (Eisenhut et al. 2008a, b). This HCR phenotype was presumably ascribed to the intracellular accumulation of toxic amounts of glycolate (Eisenhut et al. 2008a, b). It was reported that the intracellular glycolate concentration in the mutant increased to a much higher level within a few hours after the mutant was transferred from HC (5% CO_2_) to LC (air, 0.035% CO_2_) condition (Eisenhut et al. 2008a, b). Interestingly, strain WT-ΔglcD that we constructed did not exhibit the HCR phenotype (Additional file 1: Fig. S3). Further investigation suggested that strain WT-ΔglcD did accumulate intracellular glycolate, but more than 99% of glycolate was excreted to the culture (Fig. 2 and Additional file 1: Fig. S2). Glycolate excretion was previously observed in some filamentous cyanobacterial strains but not in *Synechocystis*, nor in mutant with HCR phenotype (Eisenhut et al. 2006, Eisenhut et al. 2008a, b). It is likely that glycolate excretion of strain WT-ΔglcD helped maintain the intracellular glycolate concentration at a low level, which allows the cell to grow normally at ambient CO_2_ level, without displaying the HCR phenotype. It is worthy to further investigate the underlying mechanism of glycolate excretion of strain WT-ΔglcD.

To further increase glycolate production, we identified the rate-limiting step by measuring the intracellular 2PG and glycolate concentrations of strain WT-ΔglcD. The result indicated that the conversion from 2PG to glycolate is fully active. As such, the oxygenase activity of Rubisco is the bottleneck of glycolate production, thus its activity needs to be increased. Accessibility to molecular oxygen is the prerequisite for the oxygenation reaction of Rubisco to occur. Overexpression of the native carboxysome-located 6RBC Rubisco of *Synechocystis* in strain WT-ΔglcD did not increase glycolate production, indicating that the oxygenation reaction of 6RBC Rubisco is hampered in the carboxysome which is a CO_2_-rich but O_2_-sheilding microcompartment (Price et al. 2008, Espie and Kimber 2011, Price 2011).

As compared to the carboxysome, the CO_2_ concentration in the cytosol is much lower. To provide the gradient for inward diffusion of CO_2_ and minimize its leakage from cell, cyanobacteria accumulates HCO_3_^−^ but not CO_2_ in the cytosol and maintains a chemical equilibrium in favor of HCO_3_^−^ over CO_2_ (Price et al. 2008, Price 2011, Burnap et al. 2015). Thus, the low-CO_2_-level cytosol might be a more favorable environment for the oxygenation reaction of Rubisco to occur. Additionally, as cyanobacteria performs oxygenic photosynthesis (Moroney et al. 2013), the photosynthetic evolved O_2_ from photosystem II located at the thylakoid membrane may also contribute to glycolate production. Overexpression of an exogenous Form II Rubisco located in the cytosol indeed increased glycolate production. Among the three forms of Rubisco, there are three reasons why we consider Form II Rubiscos promising candidates for glycolate production. First, the specificity of Form II Rubisco was reported to be extremely low, and thus can catalyze the oxygenation reaction more easily (Davidi et al. 2020). Second, Form II Rubisco is not packaged in the carboxysome, as they do not support the carboxysome biogenesis (Baker et al. 1998, Durao et al. 2015). Third, Form II Rubiscos are structurally simple, comprising only a large subunit and commonly forming an L_2_ or L_6_ oligomer (Davidi et al. 2020).

In this study, three Form II Rubiscos were selected and individually overexpressed in strain WT-ΔglcD. Among them, both RPE Rubisco and 4Pm Rubisco increased glycolate production irrespective of carbon supplement. Strain RPE-ΔglcD showed the highest glycolate titer of 2.8 g/L after 18 days of cultivation when supplied with 50 mM NaHCO_3_ (Fig. 3b). Remarkably, it compares favorably over the majority of products synthesized from CO_2_ in cyanobacteria as listed in Table 2 (Titer > 1 g/L). A more detailed list of products can be found in reviews referenced (Oliver et al. 2014, Lai  et al. 2015, Gao et al. 2016a, b; Knoot et al. 2018). This indicated that the deceptively wasteful and undesired oxygenase activity of Rubisco has immense yet undeveloped ability with regard to photosynthetic bioproduction application.

**Table 2:** Chemicals synthesized from CO_2_ by engineered cyanobacteria, with titers higher than 1 g/L

Product	Strain	Titer	References
Sucrose	*Synechococcus elongatus* UTEX 2973	8 g/L	Lin et al. (2020)
Trehalose	*Synechococcus elongatus* PCC 7942	5.7 g/L	Qiao et al. (2020)
Ethanol	*Synechocystis* sp. PCC 6803	5.5 g/L	Gao et al. (2012)
1-Butanol	*Synechocystis* sp. PCC 6803	4.8 g/L	Liu et al. (2019)
Glycolate	*Synechocystis* sp. PCC 6803	2.81 g/L	This study
2,3-Butanediol	*Synechococcus elongatus* PCC 7942	2.38 g/L	Oliver et al. (2013)
d-Lactic acid	*Synechocystis* sp. PCC 6803	2.17 g/L	Varman et al. (2013)
Isoprene	*Synechococcus elongatus* PCC 7942	1.26 g/L	Gao et al. (2016a, b)
1,3-Propanediol	*Synechococcus elongatus* PCC 7942	1.22 g/L	Hirokawa et al. (2017)
(R)-3-Hydroxybutyrate	*Synechocystis* sp. PCC 6803	1.84 g/L	Wang et al. (2018)
Isobutyraldehyde	*Synechococcus elongatus* PCC 7942	1.1 g/L	Atsumi et al. (2009)
Mannitol	*Synechococcus* sp. PCC 7002	1.1 g/L	Jacobsen et al. (2014)

**Fig. 6:**
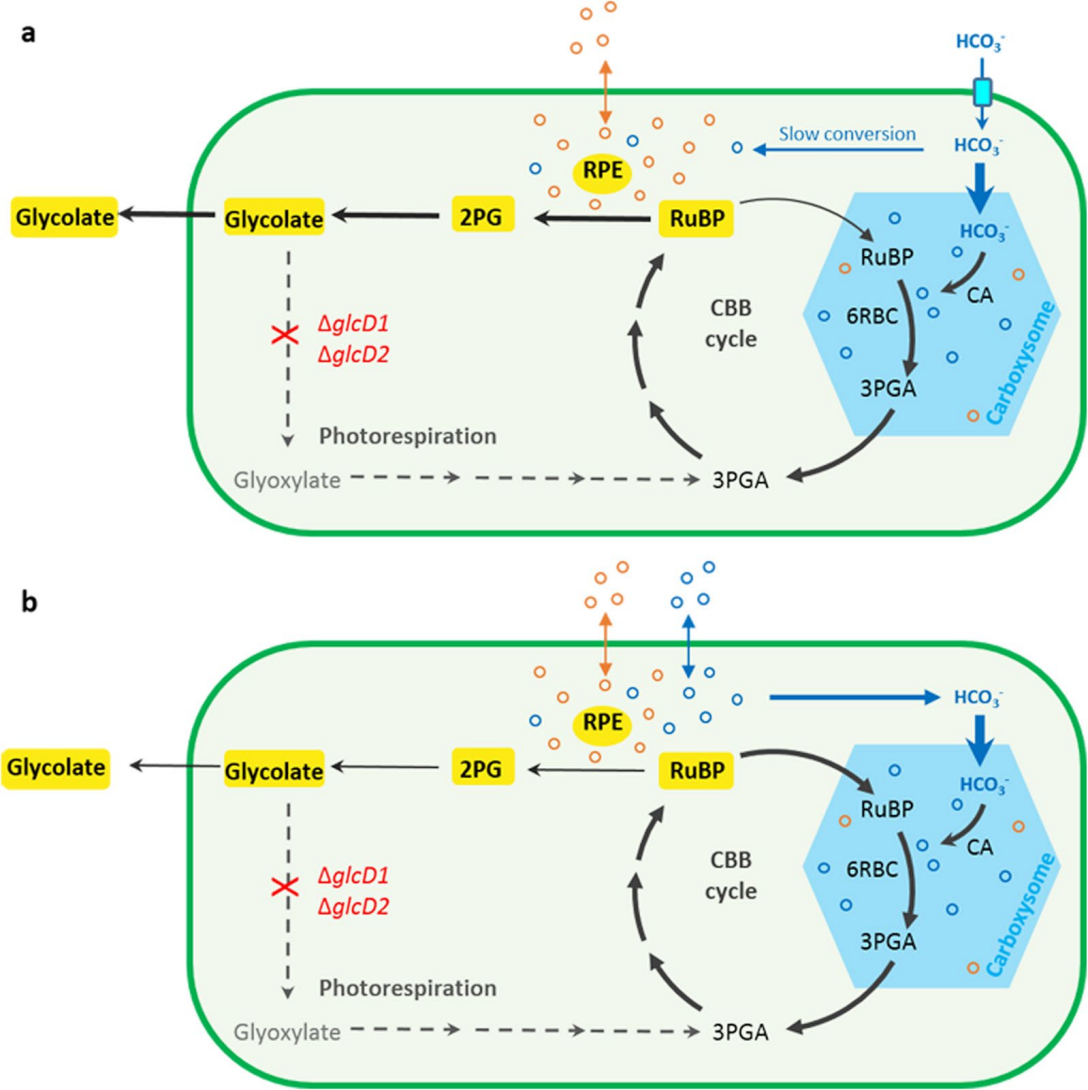
The different impacts of HCO_3_^−^ (**a**) and CO_2_ (**b**) on glycolate production by RPE-ΔglcD. The orange cycles represent molecule oxygen and the blue cycles represent CO_2_. The diffusion of O_2_ and CO_2_ are indicated by orange and blue double headed-arrows, respectively. Bold solid dark arrows indicate the direction of the favored reaction under the growth conditions

It is interesting that supply of NaHCO_3_ and CO_2_ exhibit different effects on glycolate production by strain RPE-ΔglcD, as NaHCO_3_ supply increased glycolate production while CO_2_ supply decreased glycolate production. This could be related to the different manners of HCO_3_^−^ and CO_2_ entering the cell and the CCM applied by cyanobacteria. HCO_3_^−^ is transported into the cytosol by the transporters located at the cytoplasmic membrane. The majority then enters the carboxysome and the sequestered carbonic anhydrase (CA) converts it to CO_2_. RuBP enters the carboxysome and reacts with CO_2_ catalyzed by the native Rubisco, generating two molecules of 3PGA. 3PGA escapes from the carboxysome and regenerates RuBP in the cytosol via CBB cycle (Fig. 6a). When supplied with NaHCO_3,_ the increased HCO_3_^−^ availability generally facilitates the carbon fixation of the native Rubisco in the carboxysome and results in the enhanced RuBP regeneration via CBB cycle (Fig. 6a). As regenerated in the cytosol, RuBP is preferentially oxygenated by RPE. Accordingly, less RuBP is channeled to biomass production, and the growth of strain RPE-ΔglcD is impaired upon supplementation of additional NaHCO_3_.

Furthermore, due to the absence of CA in the cytosol, the spontaneous conversion of HCO_3_^−^ to CO_2_ in the cytosol is much slower than the diffusion of CO_2_ across the cytoplasmic membrane (Mangan et al. 2016). This means that supply of NaHCO_3_ could not sharply raise the CO_2_ concentration in the cytosol. RPE Rubisco is identified from the chemolithoautotrophic symbiont in the trophosome of giant tubeworm *R. pachyptila* living at the deep-sea hydrothermal vents where the partial pressure of CO_2_ can reach up to 2.9 kPa (Lutz et al. 1994). The internal CO_2_ concentration of *R. pachyptila* can approach up to 31 mM relying on the high concentration of CA in the worm’s plume and trophosome tissue (Childress et al. 1993). Therefore, it is possible that RPE Rubisco exhibits relatively low affinity to CO_2_. Thus, the oxygenase activity of RPE is not inhibited even when supplied with 50 mM NaHCO_3_.

As an uncharged small molecule, CO_2_ can cross the cell membrane by diffusion (Price et al. 2008, Price 2011). Meanwhile, RPE Rubisco was not scattered inside the cell but aggregated near the cytoplasmic membrane. When supplied with CO_2_, the relative concentration of CO_2_ around RPE Rubisco is raised (as O_2_ concentration is not changed) (Fig. 6b). Thus, oxygenation is inhibited and carboxylation is enhanced alone with the increased availability of CO_2_. As a consequence, more RuBP is channeled to carbon fixation via CBB cycle, leading to increased cell growth and decreased glycolate production of strain RPE-ΔglcD when supplied with CO_2_ (Fig. 6b).

In summary, we demonstrated that the oxygenase function of Form II Rubisco could be explored for production of chemicals, e.g. glycolate, from CO_2_. Blocking the metabolism of photorespiration pathway led to glycolate production, and the efficiency for producing glycolate can be significantly improved when expressing Form II Rubisco in the cytosol. Thus, Form II Rubisco with distinct peculiarity can exert their versatile extraordinary capability in photosynthetic biosynthesis applications.

## Supplementary Information

**Additional file 1.**
