# Correction to: Exploring the oxygenase function of Form II Rubisco for production of glycolate from CO_2_

**DOI:** 10.1186/s13568-021-01293-7

**Published:** 2021-09-24

**Authors:** Fan Yang, Junli Zhang, Zhen Cai, Jie Zhou, Yin Li

**Affiliations:** 1 CAS Key Laboratory of Microbial Physiological and Metabolic Engineering, State Key Laboratory of Microbial Resources, Institute of Microbiology, Chinese Academy of Sciences, Beijing, 100101 China; 2 University of the Chinese Academy of Sciences, Beijing, China; 3 CAS Key Laboratory of Microbial Physiological and Metabolic Engineering, State Key Laboratory of Transducer Technology, Institute of Microbiology, Chinese Academy of Sciences, Beijing, 100101 China

## Correction to: AMB Expr (2021) 11:65 10.1186/s13568-021-01224-6

Following publication of the original article (Yang et al. 2021), the authors would like to correct the sentences in Abstract and Discussion sections. The corrections are listed below:

In Abstract section, the following sentence should be removed, “This is also the highest glycolate titer biotechnologically produced from CO_2_”.

In Discussion section, the corrected third paragraph follows:

Additionally, inactivation of glycolate metabolism was reported to render a high-CO_2_-requiring (HCR) phenotype which means the mutant was not able to grow at ambient CO_2_ level (Eisenhut et al. 2008a, b). This HCR phenotype was presumably ascribed to the intracellular accumulation of toxic amounts of glycolate (Eisenhut et al. 2008a, b). It was reported that the intracellular glycolate concentration in the mutant increased to a much higher level within a few hours after the mutant was transferred from HC (5% CO_2_) to LC (air, 0.035% CO_2_) condition (Eisenhut et al. 2008a, b). Interestingly, strain WT-ΔglcD that we constructed did not exhibit the HCR phenotype (Additional file 1: Fig. S3). Further investigation suggested that strain WT-ΔglcD did accumulate intracellular glycolate, but more than 99% of glycolate was excreted to the culture (Fig. 2 and Additional file 1: Fig. S2). Glycolate excretion was previously observed in some filamentous cyanobacterial strains and green alga like *Chlamydomonas* (Eisenhut et al. 2006; Günther et al. 2012, 2018). It is reported that Chlamydomonas could be forced to produce and excrete glycolate constantly without negative impact on cell vitality under specific conditions (Günther et al. 2012; Taubert et al. 2019). A glycolate titer of 3.1 g/L within 21 days was achieved by the aeration of a mixture of 40% O_2_/0.2% CO_2_ and by the addition of EZA (6-Ethoxy-2-benzothiazolesulfonamide), an efficient inhibitor for both CCMs and the GlyDH (glycolate dehydrogenase) in C2 cycle (Taubert et al. 2019). However, glycolate excretion was not observed in *Synechocystis*, nor in mutant with HCR phenotype (Eisenhut et al. 2006, 2008a, b). It is likely that glycolate excretion of strain WT-ΔglcD helped maintain the intracellular glycolate concentration at a low level, which allows the cell to grow normally at ambient CO_2_ level, without displaying the HCR phenotype. It is worthy to further investigate the underlying mechanism of glycolate excretion of strain WT-ΔglcD.
