# Supplementary material for: Exploring the oxygenase function of Form II Rubisco for production of glycolate from CO2
Source: AMB Express. 2021 May 8;11:65. doi: 10.1186/s13568-021-01224-6 (PMC8106553; doi:10.1186/s13568-021-01224-6)
Supplement: Supplementary file 1 — Additional file 1. [file 13568_2021_1224_MOESM1_ESM.docx]

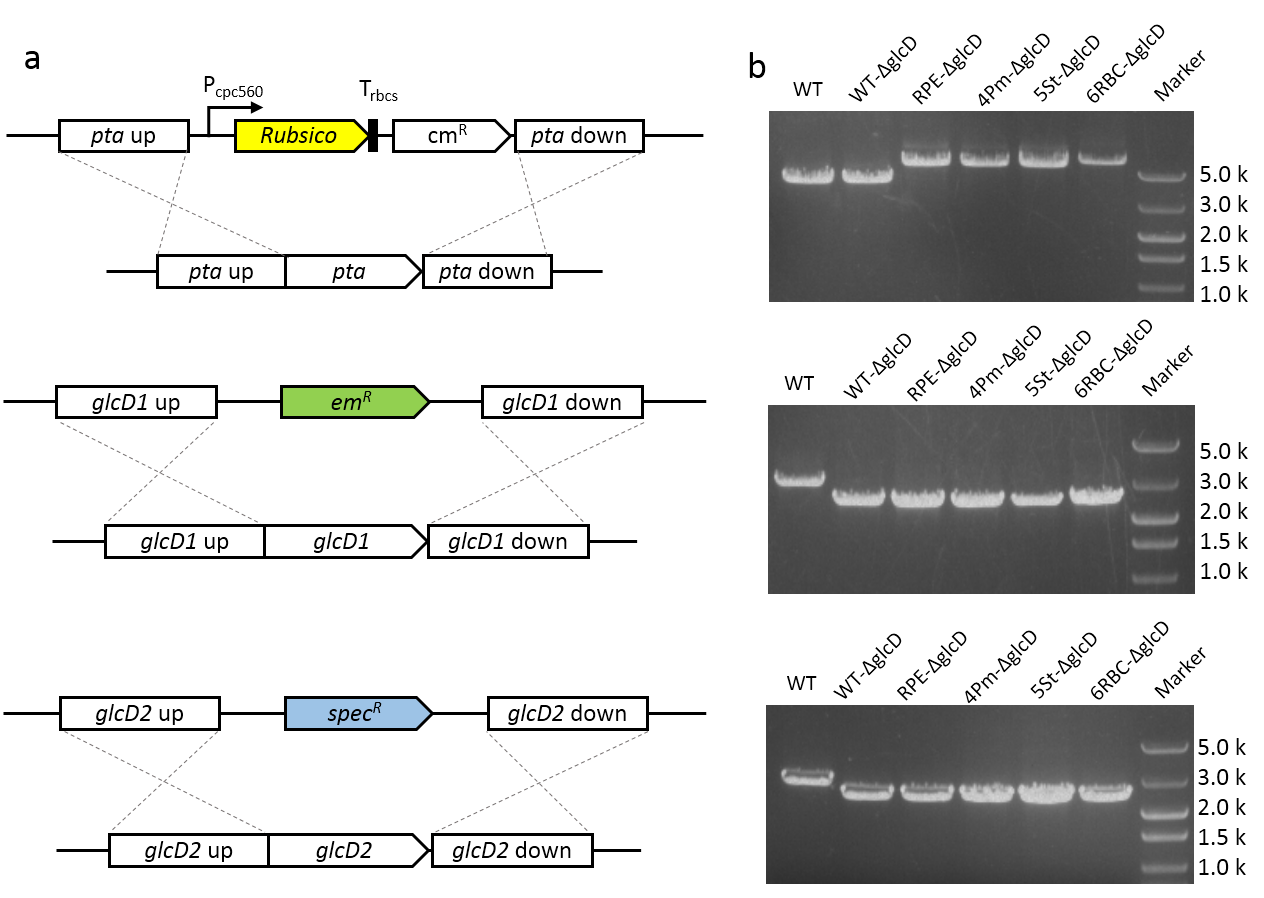


**Fig. S1** The genetic modifications in glycolate-producing strains. (a) Genetic modifications. (b) Colony PCR verification of the recombinant cassette at the sites of *pta*, *glcD1* and *glcD2*. The specific primers located at the upstream and downstream of the recombinant cassette were used to confirm the complete segregation of all mutants.


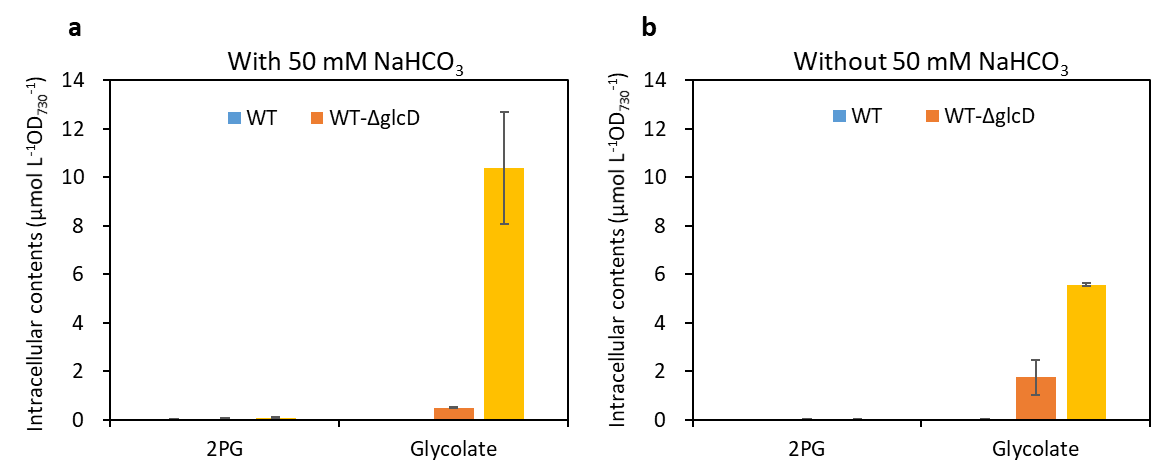


**Fig. S2** The intracellular contents of 2PG and glycolate in strains WT, WT-ΔglcD and RPE-ΔglcD. Samples were taken after 3 days cultivation with (a) or without (b) different concentration of NaHCO_3_ under 100 μmol photons m^-2^s^-1^ light intensity.


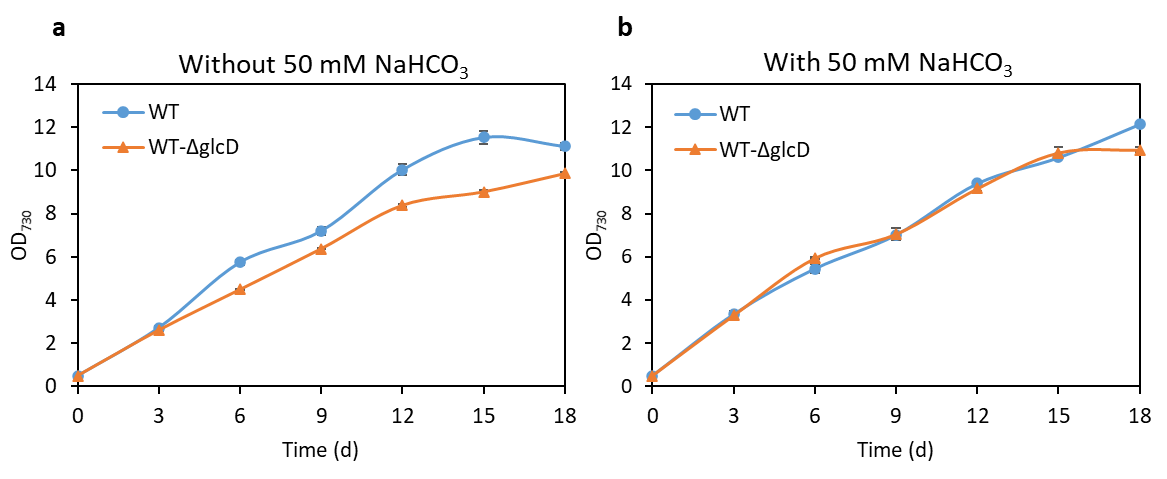


**Fig. S3** Growth profiles of the WT strain and strain WT-ΔglcD without (a) and with (b) 50 mM NaHCO_3_. Cells were cultivated at 30 ^o^C under 100 μmol photons m^-2^s^-1^ light intensity. Error bars represent standard deviations from biological triplicates conducted in three independent experiments.


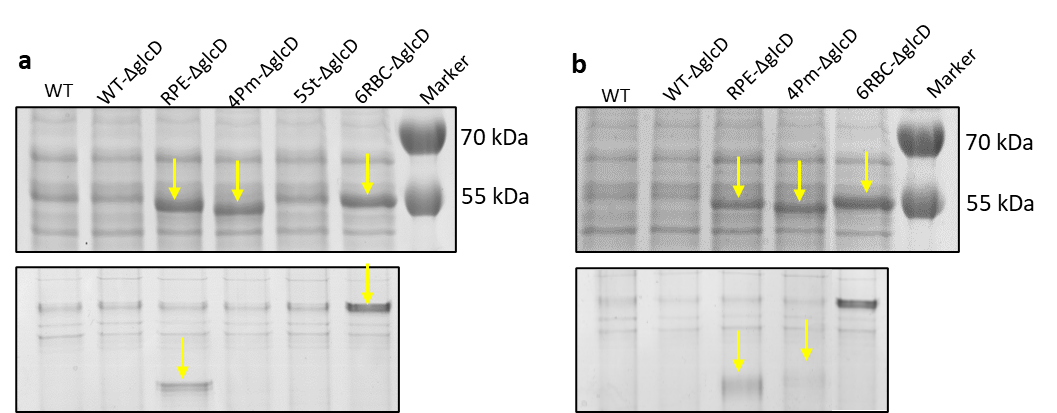


**Fig. S4** The protein expression analysis of Form II Rubisco proteins without (a) or with (b) 50 mM NaHCO_3_. The expression (up) and assembly (down) level of Form II Rubiscos under both conditions were detected with SDS-PAGE and Native-PAGE respectively. The yellow arrows indicate the target protein bands.

**Table S1 Strains and Plasmids Used in This Study.**

| **Strains and plasmids** | **Relevant characteristics** | **Reference** |
| --- | --- | --- |
| **Strains** |  |  |
| *E. coli* DH5α | Commercial strain for plasmids construction | Lab storage |
| *Synechocystis* sp. PCC 6803 | Wild type | Lab storage |
| WT-△glcD | *glcD1*::*em^r^*; *glcD2*::*spec^r^* | This work |
| RPE-△glcD | *pta*::P_cpc560_-*rpe*-T_rbcs_-*cm^r^*  *glcD1*::*em^r^*; *glcD2*::*spec^r^* | This work |
| 4Pm-△glcD | *pta*::P_cpc560_-*4pm*-T_rbcs_-*cm^r^*  *glcD1*::*em^r^*; *glcD2*::*spec^r^* | This work |
| 5St-△glcD | *pta*::P_cpc560_-*5St*-T_rbcs_-*cm^r^*  *glcD1*::*em^r^*; *glcD2*::*spec^r^* | This work |
| 6Rbc△glcD | *pta*::P_cpc560_-*6rbc*-T_rbcs_-*cm^r^*  *glcD1*::*em^r^*; *glcD2*::*spec* | This work |
| RPE-GFP | *pta*::P_cpc560_-*rpe-gfp*-T_rbcs_-*cm^r^* | This work |
| 6RBC-GFP | *pta*::P_cpc560_-*6rbcL-gfp*-*6rbcS*-T_rbcs_-*cm^r^* | This work |
| **Plasmids** |  |  |
| pUC57-pta | pUC57 derivate, Amp^r^ Cm^r^, containing *pta* knockout cassette | This work |
| pUC57-glcD1 | pUC57 derivate, Amp^r^ Em^r^, containing *glcD1* knockout cassette | This work |
| pUC57-glcD2 | pUC57 derivate, Amp^r^ Spec^r^, containing *glcD2* knockout cassette | This work |
| pta-RPE | pUC57-pta derivate, containing P_cpc560_-*rpe*-T_rbcs_ expression cassette | This work |
| pta-4Pm | pUC57-pta derivate, containing P_cpc560_-*4pm*-T_rbcs_ expression cassette | This work |
| pta-5St | pUC57-pta derivate, containing P_cpc560_-*5st*-T_rbcs_ expression cassette | This work |
| pta-6RBC | pUC57-pta derivate, containing P_cpc560_-*6rbcL*-*6rbcS*-T_rbcs_ expression cassette | This work |
| pta-RPE-GFP | pUC57-pta derivate, containing P_cpc560_-*rpe-gfp*-T_rbcs_ expression cassette | This work |
| pta-6RBC-GFP | pUC57-pta derivate, containing P_cpc560_-*6rbcL-gfp*-*6rbcS*-T_rbcs_ expression cassette | This work |

**Table S2 Primers Used in This Study.**

| **Primers** | **Sequence (5’-3’)** |
| --- | --- |
| ptaCm-1 | cgacgttgtaaaacgacggccagtgaattccgggaaggggtaggcattac |
| ptaCm-2 | gttcattatcaactaattgacaattgacaattccccac |
| ptaCm-3 | caattgtcaattagttgataatgaactgtgctg |
| ptaCm-4 | attaagagtaaaaatcgaatttctgccattc |
| ptaCm-5 | gcagaaattcgatttttactcttaattagttaaaatgatcc |
| ptaCm-6 | aacagctatgaccatgattacgccaagcttttggctttatttcccgatac |
| RPE-1 | acgttgaggttatttaaattgagctcacctgtagagaagagtccctgaatatcaaaatggtggg |
| RPE-2 | tgaattaatctcctacttgac |
| RPE-3 | taaagtcaagtaggagattaattcaatggcattagaccaaaccaatcg |
| RPE-4 | gtaattgccaaaactgtaactcaaggatccttacttgtgtacgcccag |
| 4Pm-1 | acgttgaggttatttaaattgagctcacctgtagagaagagtccctgaatatcaaaatggtggg |
| 4Pm-2 | tgaattaatctcctacttgac |
| 4Pm-3 | taaagtcaagtaggagattaattcaatggaccagtcttctcgttac |
| 4Pm-4 | gtaattgccaaaactgtaactcaaggatccttagtcgatacccagacg |
| 5St-1 | acgttgaggttatttaaattgagctcacctgtagagaagagtccctgaatatcaaaatggtggg |
| 5St-2 | tgaattaatctcctacttgac |
| 5St-3 | taaagtcaagtaggagattaattcaatgtcccttgaccaaacc |
| 5St-4 | gtaattgccaaaactgtaactcaaggatccttatttgtgcacgcccag |
| 6Rbc-1 | acgttgaggttatttaaattgagctcacctgtagagaagagtccctg |
| 6Rbc-2 | tgaattaatctcctacttgac |
| 6Rbc-3 | taaagtcaagtaggagattaattcaatggtacaagccaaagcag |
| 6Rbc-4 | taatctcctacttgactttttagagggtatccatggc |
| 6Rbc-5 | ggataccctctaaaaagtcaagtaggagattaattcaatgaaaactttacccaaagagc |
| 6Rbc-6 | gtaattgccaaaactgtaactcaaggatccttagtaacggccttggttttg |
